# Can Lighting Influence Self-Disclosure?

**DOI:** 10.3389/fpsyg.2017.00234

**Published:** 2017-02-23

**Authors:** Veli Mehta, Sumitava Mukherjee, Jaison A. Manjaly

**Affiliations:** ^1^Centre for Cognitive Science, Indian Institute of Technology GandhinagarGandhinagar, India; ^2^Humanities and Social Sciences, Indian Institute of Management IndoreIndore, India

**Keywords:** ambient brightness, environment, self-disclosure, perceptual metaphors, null effects

## Abstract

With the advent of social networks where people disclose a lot of their information and opinions publicly, this research attempted to re-look at the effect of environmental lighting on willingness and actual disclosure of personal information. Previous literatures mostly addressed counseling setups and the findings were mixed. In order to clarify the effect of lighting on self-disclosure, two experiments were conducted with reported willingness to disclose (Experiment 1) as well as actual disclosure (Experiment 2) on a range of topics like social issues, body, money, work, and personality. While quite a handful of studies have reported differences in disclosure from very subtle environmental lighting manipulations, in both experiments we could not find any effect of ambient room lighting conditions on self-disclosure. These results call for caution both in over-interpreting subtle environmental effects and in increased generalization of perceptual metaphors to actual behavior.

“Nothing is hidden except for the purpose of having it revealed, and nothing is secret except for the purpose of having it come to light”

- Bible (Mark 4:22)

## Introduction

We often hear phrases like “dark secrets” or “bringing to light” which suggest a metaphorical relation between darkness and secrecy or privacy of information. It is natural for darkness to conceal one’s identity, because dark environments reduce possibility of identification. However, a stronger claim, based on the idea of embodied cognition (Wilson and Foglia, 2011) was made where it was argued that people could also generalize the phenomenal experience of darkness, in-turn expecting others would have difficulty in seeing them and hence disclose more (Zhong et al., 2010). Empirically, a dark room or simply wearing dark sunglasses were purported to give rise to actual anonymity and a feeling of “illusory anonymity” that consequently affected information disclosure and related behavior (Zhong et al., 2010). How effective are subtle environmental lighting manipulations to change self-disclosing behavior? Does the mind always respond to perceptual metaphors that make people reveal more when there is darkness?

Effects of lighting on disclosure behaviors in social and offline contexts have been investigated through several experiments but these have yielded mixed results. In an early study on the effect of lighting conditions on disclosure (Carr and Dabbs, 1974), it was shown that dim conditions were rated as more intimate compared to bright conditions and this led to an increase in latency to talk in dim compared to bright conditions to counter the level of intimacy introduced by the dim condition. Chaikin et al. (1976) also showed greater degree of disclosure amongst participants in intimate compared to non-intimate settings. Non-intimate settings included, among other things, an overhead direct florescent light and intimate settings included, among other things, indirect lighting through a floor lamp and a small table lamp. Similarly, Bille’s (2015) ethnographic account revealed the role of light in bringing about experiences of community, connection and intimacy amongst neighborhood residents.

However, when Lecomte et al. (1981) manipulated lighting, distance and amount of time for interaction, they did not find any significant effect of lighting on client disclosure. Further, Gifford (1988) found that more intimate communication takes place in bright compared to dim lighting conditions. In yet another study (Okken et al., 2013) investigating the level of disclosure between patient and physician, it was found that in situations of high perceived threat (to health), brightly lit consulting rooms elicited greater positive affective experiences and greater intended self-disclosure due to an increase in perceived spaciousness caused by brightness of light.

Some studies have also made attempts to elucidate the mechanisms by which the effects of lighting environments on self-disclosure can be explained. Dim lighting played a role in producing relaxation and positive emotions (Flynn et al., 1973; Flynn, 1988; Baron et al., 1992). Participants rated dimly lit dining areas to inspire greater sociability, positive emotion as well as behavioral intention compared to brightly lit dining areas while dining with a special friend (Wardono et al., 2012). A study by Miwa and Hanyu (2006) showed that dim lighting lead to more pleasant and relaxed feelings in the participants leading to greater disclosure. Indeed, it has been suggested that dim lighting does result in a positive mood (Baron et al., 1992) and positive mood could lead to greater disclosure (Forgas, 2011). A similar study (Stefanone et al., 2009) indicated that people who perceive their environment to be warm and pleasant tend to disclose more over web-blogs. These findings claim that positive, warm and relaxing feeling could be the mediating factor that brings about increased disclosure in presence of dim lighting conditions. However, the dynamics behind the effect of lighting conditions on self-disclosure may not be that straightforward. For instance, Steidle and Werth (2014) have reported that people tend to have greater self-awareness and tend to exert greater extent of reflective self-control when exposed to bright compared to dim environments and did not find effects of lighting on perceived anonymity. Thus, there seem to be multiple possible explanations for the observed effect of different lighting conditions on self-disclosure.

Effects of lighting on self-disclosure are far from conclusive. There are a couple of ways in which the discrepancies in the results could be understood. First, the independent manipulations and context in most of the studies were counseling or medical settings and hence there might be additional variables that add variability to the data like client–counselor relationship, décor, trust, gender, or perceived health threats, etc. These studies have hardly investigated the effect of lighting condition in isolation. Another important concern is the dependent measure that ranged from sentences spoken to ratings. Additionally, several mechanisms have been suggested like positive mood (Miwa and Hanyu, 2006), perceived spaciousness (Okken et al., 2013), self-awareness, self-control (Steidle and Werth, 2014), and perceived anonymity (Zhong et al., 2010) which make it complicated to predict effects in future studies.

The experiments in this paper addressed the issue of lighting on self-disclosure measured in two different ways: the willingness to disclose (Experiment 1) and actual disclosure (Experiment 2). We used the context of online self-disclosure in the cover story given the commonality of self-disclosure in social media. With increasing social networking, personal and private information are available for public access, often as a result of self-disclosure. Given conflicting findings from the past literature (e.g., Lecomte et al., 1981; Gifford, 1988; Okken et al., 2013) we took the role of a skeptic to find whether there is a significant effect of ambient lighting environments.

## Experiment 1: Willingness to Disclose

### Participants

Fifty-seven students from Indian Institute of Technology at Gandhinagar, Gujarat (age = 18–27 years, males = 46, females = 11) participated in the experiment voluntarily. A formal institutional review board was not present when data was initially collected and hence we used guidelines from the Helsinki declaration of ethics for human behavioral studies. Participation was voluntary with the option of exiting the study at any point in time.

### Method

This experiment was conducted individually in a room that contained a single table and a chair so that participants could comfortably write their responses. Two kinds of lighting fixtures were used to manipulate the amount of lighting in the room. A tubular florescent lamp (OSRAM T5, 28 W) was turned on during the ‘Bright’ lighting condition and a CFL (Lighto, 5 W CFL) was turned on during the ‘Dim’ lighting condition. Participants were randomly assigned to either the Bright or Dim lighting conditions. The ambient light in the room during both the conditions was measured using a Luxmeter (Seconic L-308S) by placing it horizontally on the table where participants were expected to place their questionnaires and fill them up. The ambient light was recorded as 40 lux during the ‘Dim’ condition and 372 lux in the ‘Bright’ condition (see Images 1 and 2 in Supplementary Material for snapshots of the room under different lighting conditions).

The experiment was conducted in two parts. During the first part, participants were told to rate the extent to which they would talk about certain kinds of information on a social networking site that is publicly accessible. Ratings were from 1 to 7 (1 = least willing to disclose and 7 = most willing to disclose). Thirty items were selected from Jourard’s Self Disclosure Questionnaire (Jourard and Lasakow, 1958) related to a range of attitudes toward social issues, body, money, work, and personality (see Table 1 in Supplementary Material for all items). Sample items were “How I wish I looked; my ideas for overall appearance.” and “Whether or not I have savings and the amount of my savings.” The items were printed on a paper on which they had to rate their willingness to talk about each of them. The questionnaire was given to participants who sat alone in the room to fill it up.

After the participant completed the questionnaire, the person walked to the experimenter who was waiting outside and was handed over a ‘feedback form’. The feedback form was intended to get feedback about the room that was being used for the current study. Among the different environmental and affective variables suggested in previous literatures, we included perceived anonymity, self-awareness, affective experience, perceived spaciousness and perceived threat. The following items were used: (i) two items related to Perceived Anonymity adapted from Zhong et al. (2010); (ii) one item each relating to self-awareness about surroundings, public self-awareness and private self-awareness selected from the Situational Self Awareness Scale (Govern and Marsch, 2001) based on the loading factors, (iii) two items measuring Perceived spaciousness adapted from Okken et al. (2013); (iv) three items measuring affective experience adapted from Okken et al. (2013), and (v) one item measuring perceived threat (‘While doing the task, I felt a threat to my privacy and social image’). These items were intended to measure environmental and affective states that might be affected by environmental lighting as suggested by previous researchers. All items were to be rated on seven point bipolar scales from ‘Strongly Disagree’ to ‘Strongly Agree’. Finally, participants were asked to rate the extent of perceived lighting of the room on a seven point bipolar scale from Dim to Bright. The complete list of items used in the feedback form has been given in Table 3 of Supplementary Material. Participants were debriefed at the end of the study.

### Results

Lighting was perceived to be less in the dim condition (*M* = 3.04 and *SD* = 1.11) compared to the bright condition (*M* = 4.67 and *SD* = 1.24), showing that our manipulation of luminance worked (Mann–Whitney *U* = 130, *n*_1_ = 28, *n*_2_ = 27, and *p* = 0.0001, two-tailed). The 30 items from the Self-Disclosure questionnaire were found to be reliable for the present study (α = 0.88) and hence the responses of each of the participants on all the 30 questions were averaged to achieve a representative score for willingness to self-disclose. One participant from the Dim condition and one participant from the Bright condition were removed from analysis, as the age was an outlier (>2.5 *SD*). Willingness to self-disclose in the ‘Bright’ condition (*M* = 3.89 and *SD* = 0.77) was similar to the ‘Dim’ condition (*M* = 3.76 and *SD* = 0.95), *F*(1,53) = 0.323, *p* = 0.57, and $\eta_{p}^{2}$ = 0.006^1^.

Next, we examined whether any of the affective variables were reported to be different by participants in the dim versus bright groups. **Table 1** provides information about differences between two groups with respect to environmental and affective variables.

**Table 1:** Mean and standard deviation (*SD*) of responses to all the feedback items for both conditions measured in Experiment 1 along with results of Mann–Whitney *U*-test for each comparison.

S. No.	Variable measured	Mean (*SD*): Dim	Mean (*SD*): Bright	Analysis
1.	Perceived Anonymity	4.07 (1.51)	3.81 (1.79)	Mann–Whitney *U* = 355.0, *n*_1_ = 28, *n*_2_ = 27, *p* = 0.69, two-tailed.
2.	Environmental Self Awareness	5.00 (1.82)	4.85 (1.74)	Mann–Whitney *U* = 232.5, *n*_1_ = 28, *n*_2_ = 27, *p* = 0.66, two-tailed
3.	Public Self Awareness	1.85 (1.11)	3.37 (2.15)	Mann–Whitney *U* = 232.5, *n*_1_ = 28, *n*_2_ = 27, and *p* = 0.01^∗^, two tailed
4.	Private Self Awareness	5.50 (1.42)	5.62 (0.88)	Mann–Whitney *U* = 371, *n*_1_ = 28, *n*_2_ = 27, and *p* = 0.89, two-tailed
5.	Affective experience	5.08 (1.38)	5.09 (1.18)	Mann–Whitney *U* = 367.0, *n*_1_ = 28, *n*_2_ = 27, and *p* = 0.85, two-tailed.
6.	Perceived Threat	2.14 (1.43)	2.74 (1.76)	Mann–Whitney *U* = 299, *n*_1_ = 28, *n*_2_ = 27, and *p* = 0.17, two-tailed
7.	Perceived Spaciousness	2.67 (1.33)	2.94 (1.63)	Mann–Whitney *U* = 346.5, *n*_1_ = 28, *n*_2_ = 27, and *p* = 0.59, two-tailed

*^∗^Statistically significant effect.*

As shown in **Table 1**, public self-awareness was significantly lower (Mann–Whitney *U* = 232.5, *n*_1_ = 28, *n*_2_ = 27, and *p* = 0.01, two tailed) in the dim (*M* = 1.86 and *SD* = 1.11) compared to the bright condition (*M* = 3.37 and *SD* = 2.15). No other effect of two lighting conditions was found on environmental or affective variables. We explored if the effect of lighting on willingness to disclose could be indirect, i.e., whether lighting environment could influence willingness to disclose information through its impact on self-awareness by conducting a Path Analysis (see Table 1 in Supplementary Material). This analysis also did not reveal an effect of lighting on willingness to disclose through a mediating effect of self-awareness.

Finally, an ANCOVA was calculated with gender as a covariate, on willingness to disclose ratings. This analysis also did not show any significant effect of lighting conditions on willingness to disclose, *F*(1,52) = 0.308, *p* = 0.58, and $\eta_{p}^{2}$ = 0.006. These results clearly show that neither did lighting conditions have any effect on willingness to disclose nor were any affective or environmental variables significantly affected by the ambient lighting conditions.

## Experiment 2: Actual Disclosure

We used the same items but asked participants to voluntarily disclose their attitudes and information for each of those items to check if the effects are similar for actual disclosure compared to willingness to disclose.

### Participants

Fifty-six students having demographic characteristics similar to those who participated in the previous study (age = 18–25 years, males = 47, and females = 9) participated voluntarily.

### Method

The same room with similar ambient lighting was used with 40 lux in the ‘Dim’ condition and 372 lux in the ‘Bright’ condition (see Supplementary Material for snapshots of the room under different lighting conditions).

As in Experiment 1, in the first part, participants were told that we were aggregating information that may be shared with an online social networking site. They were given the list of all items and were told that it was not compulsory for them to answer all questions. We also instructed them to specifically write ‘I don’t want to share’ if they did not intend to share that information and write ‘I don’t know’ or ‘I don’t understand’ if such be the case. It was mentioned that they should be honest and write the first thing that comes to their mind without thinking too much. The questionnaire was then given to them and the experimenter left the room. The participant sat alone in the room to fill it up. The questionnaire contained the same 30 questions, adapted from the items of self-disclosure questionnaire used in Experiment 1. After the participant completed the questionnaire, the person walked to the experimenter who was waiting outside and was handed over a ‘feedback form’. This Feedback form was similar to the one used in the previous study.

### Results

Lighting was perceived to be less in the dim condition (*M* = 2.71 and *SD* = 0.81) compared to the bright condition (*M* = 4.68 and *SD* = 0.98), confirming our manipulation, Mann–Whitney *U* = 46, *n*_1_ = 28, *n*_2_ = 28, and *p* = 0.0001. Each answer to an item was scored as ‘1’ if it was responded to or as ‘0’ if it was stated “I don’t want to share”. This total score calculated out of 30 was the dependent measure for actual disclosure. The mean number of items responded to in Dim (*M* = 28.71 and *SD* = 2.54) and Bright conditions (*M* = 28.79 and *SD* = 2) was almost similar (See Table 2 in Supplementary Material for mean and percentage of items responded to). There was no effect of lighting on actual disclosure (Mann–Whitney *U* = 368, *n*_1_ = *n*_2_ = 28, and *p* = 0.67, two-tailed)^2^. In order to control for the possibility of most individuals having responded with ‘I don’t know’ as an answer more frequently, a new measure for Actual disclosure was calculated by following formula:

$$\mathrm{Actual}\;\mathrm{Disclosure}=\frac{\mathrm{Total}\;\mathrm{questions}\;\mathrm{responded}\;\mathrm{to}\;\mathrm{without}\;‘I\;don't\;\mathrm{want}\;\mathrm{to}\;share'}{30-\mathrm{Total}\;\mathrm{questions}\;\mathrm{with}\;‘I\;don't\;know'\;\mathrm{as}\;\mathrm{response}}\times 100$$

Even when this new measure of Actual Disclosure was considered, there was no effect of lighting on actual disclosure (Mann–Whitney *U* = 374.5, *n*_1_ = *n*_2_ = 28, and *p* = 0.76, two-tailed)^3^.

Next, we examined whether any of the affective variables were reported to be different by participants in the dim versus bright groups.

As seen in **Table 2**, there was no significant difference between the dim (*M* = 4.0 and *SD* = 1.53) and bright (*M* = 3.9 and *SD* = 1.72) conditions with respect to perceived anonymity; (Mann–Whitney *U* = 383.0, *n*_1_ = *n*_2_ = 28, and *p* = 0.88, two tailed). Even though this finding was at odds with that reported by Zhong et al. (2010), it was in line with that reported by Steidle and Werth (2014). Also, participants in the bright condition (*M* = 2.3 and *SD* = 1.26) reported significantly lesser (Mann–Whitney *U* = 255.5, *n*_1_ = *n*_2_ = 28, *p* = 0.02 two-tailed) perceived spaciousness compared to those in the dim condition (*M* = 3.2 and *SD* = 1.54). This result was found to be at odds from that as reported by Okken et al. (2013). No other effect of two lighting conditions was found on environmental or affective variables.

**Table 2:** Mean and *SD* of responses to all the feedback items for both conditions measured in Experiment 2 along with results of Mann–Whitney *U*-test for each comparison.

S. No.	Variable measured	Mean (*SD*): Dim	Mean (*SD*): Bright	Analysis
5.	Perceived Anonymity	4.0 (1.53)	3.96 (1.72)	Mann–Whitney *U* = 383.0, *n*_1_ = *n*_2_ = 28, and *p* = 0.88, two-tailed
6.	Environmental Self Awareness	4.89 (1.59)	4.61 (2.13)	Mann–Whitney *U* = 384.5, *n*_1_ = *n*_2_ = 28, and *p* = 0.90, two-tailed
7.	Public Self Awareness	2.36 (1.59)	2.35 (1.39)	Mann–Whitney *U* = 379, *n*_1_ = *n*_2_ = 28, and *p* = 0.82 two-tailed
8.	Private Self Awareness	5.86 (1.14)	5.71 (1.01)	Mann–Whitney *U* = 345.5, *n*_1_ = *n*_2_ = 28, and *p* = 0.41, two-tailed
13.	Affective experience	5.09 (1.26)	5.2 (1.14)	Mann–Whitney *U* = 377.0, *n*_1_ = *n*_2_ = 28, and *p* > 0.81, two-tailed
14.	Perceived threat	2.79 (1.83)	2.32 (1.63)	Mann–Whitney *U* = 336.5, *n*_1_ = *n*_2_ = 28, and *p* = 0.35, two-tailed
17.	Perceived spaciousness	3.2 (1.54)	2.32 (1.49)	Mann–Whitney *U* = 255.5, *n*_1_ = *n*_2_ = 28, and *p* = 0.02^∗^, two-tailed

*^∗^Statistically significant effect.*

Further an ANCOVA was conducted with Gender as a covariate and Actual Disclosure (using the new formula) as the dependent variable and lighting conditions as a fixed factor. This analysis also did not lead to an effect of lighting conditions on actual disclosure, *F*(1,53) = 0.003, *p* = 0.956, and $\eta_{p}^{2}$ = 0.0001. The overall results showed hardly any influence of lighting either on disclosure or on any of the affective variables.

## Discussion

One arbitrator of empirical uncertainties is a replication attempt. Here, we tried to conceptually replicate the idea that dim environments make people disclose more about themselves. However, two experiments did not find any evidence for such a claim. Experiment 1 involved ratings of willingness to disclose a variety of attitudes and information on social networking sites that implied online disclosure in front of strangers or acquaintances. It was found that willingness to disclose information about self was not influenced by lighting conditions. Indirect effects of lighting on willingness to disclose information about oneself were also not found. In Experiment 2, we asked people to actually disclose (voluntarily) their information for the same list of items as in Experiment 1 and found no effect of lighting on actual disclosure. We also did not find any significant differences on almost all the affective/environmental variables asked at the end of both studies; which also show that such affective states are contextually sensitive. Self-disclosure is a complex measure and can be modified by the cover story of experiments, context, experimenter, motivational goals, and a multitude of related variables. Thus, while we were not able to find any effect of lighting on the Jourard’s Self Disclosure scale, we do not claim that no measure will be affected by lighting conditions. Of course, the evidence is more weighted towards a null effect of lighting conditions. Also, considering the fact that data pertaining to most of the variables violated the assumption of normality in the current studies we would need to use much larger sample sizes in future studies.

One possible reason for not tapping differences in affective states could probably be a large variance in population, context and adaptability in different experiments which further echoes the concern regarding generalization of apparent underlying mechanisms related to luminance. Another concern could be the levels of luminescence selected for the current studies. The accepted standards for Dim and Bright luminescence at workplaces are 150 lux and 1500 lux, respectively (as suggested by Steidle and Werth, 2014). However, we used lighting levels where dim was 40 lux and bright was 372 lux. These levels of lighting were chosen as they were considered apt for hostel rooms or corridors where most of the interactions are likely to take place amongst students (which our sample was made of). Moreover, lighting environments of 40 lux were rated as significantly dimmer compared to lighting environments of 372 lux by participants. A third concern is the dependent variable. It is plausible that the items used in our study had little variance and hence were not conducive to priming effects. We do note a ceiling effect in Experiment 2 with most participants opting to disclose the information on almost all items. This can be further colored by the participant pool (here, college students) but again, this is similar to many previous studies.

More broadly, the idea that affective contextual evaluations are often represented implicitly in perceptual metaphors (Lakoff and Johnson, 1999), and that can in turn influence judgments and decisions (Lee and Schwarz, 2014) has been on the rise. Based on such common associations, some studies have found that experiences of darkness can increase self-disclosure while others have not been able to find such an effect. One major motivation for the current study was to shed more light on some previous discrepant findings. Our results dovetail with the results of Lecomte et al. (1981), thus yielding more empirical evidence in support of the finding that subtle environmental factors like lighting *do not* have effects on information disclosure. It should be noted that studies in which effects of lighting conditions on self disclosure have been reported (e.g., Carr and Dabbs, 1974; Chaikin et al., 1976; Miwa and Hanyu, 2006; Okken et al., 2013) also involved variability in client–counselor relationship, décor, trust, gender, or perceived health threats in their experimental settings. The current studies were attempts at studying effects of lighting on disclosure in isolation. We also ensured that other environmental factors do not confound the effect of luminance.

Care and caution is thus called in deciding whether lighting influences self-disclosure. Additionally, we need to be sensitive to the larger problem of false-positives and publication-bias dominant for studies that report statistically positive effects of subtle ambient light conditions. Priming and contextual effects are in general highly sensitive and might not always replicate which suggest that researchers themselves must provide evidence of direct replications (Cesario, 2014). Hence, it is important for previous researchers who have reported effects of lighting on disclosure to revisit their findings and gage the stability of the effect. Overall, we find limited support for the idea that perceptual metaphors guide unconscious guidance systems via subtle lighting manipulations in the domain of self-disclosure.

## Ethics Statement

We used guidelines from the Helsinki declaration of ethics for human behavioral studies. Subjects were approached randomly and asked if they would volunteer to participate. Those who agreed had signed up on a sheet that stated that they were willing to participate voluntarily.

## Author Contributions

All authors were involved in planning the study. VM collected the data and performed the analysis, SM and VM drafted the manuscript and JM suggested revisions.

## Conflict of Interest Statement

The authors declare that the research was conducted in the absence of any commercial or financial relationships that could be construed as a potential conflict of interest.

## Footnotes

1 Mann—Whitney *U* = 366.5, *n*_1_ = 28, *n*_2_ = 27, and *p* = 0.846, two-tailed. Mann–Whitney *U* is a non-parametric test used to test significance of difference between two-independent samples. This test was used for all other comparisons pertaining to affective and environmental variables, as for those variables, assumptions of normality were not satisfied.

2 Mann–Whitney *U* is a non-parametric method of testing for significance of difference between two-independent samples. The Actual disclosure scores for both the groups were not normally distributed and so, Mann–Whitney *U* was calculated instead of one way ANOVA, as the assumption of normality was not satisfied. On calculating ANOVA with the some data, we get the following result: *F*(1,54) = 0.014, *p* = 0.907, and $\eta_{p}^{2}$ = 0.0001.

3 The Actual Disclosure scores according to the new measure for both the groups were not normally distributed and so, Mann–Whitney *U* was calculated instead of one way ANOVA, on calculating ANOVA with the some data, we get the following result: *F*(1,54) = 0.008 and *p* = 0.928, $\eta_{p}^{2}$ = 0.0001.
