# Supplementary material for: Can Lighting Influence Self-Disclosure?
Source: Front Psychol. 2017 Feb 23;8:234. doi: 10.3389/fpsyg.2017.00234 (PMC5322203; doi:10.3389/fpsyg.2017.00234)
Supplement: Supplementary file 1 [file Table_1.DOCX]

Can lighting influence self-disclosure?

SUPPLEMENTARY MATERIAL

Table 1: Mean values for willingness to disclose on the items from Jourard’s Self Disclosure scale used in Experiment 1 for Dim and Bright Room lighting conditions.

| Sr. no. | Statements | Mean (SD) Dim | Mean (SD) Bright |
| --- | --- | --- | --- |
| 1 | How I wish I looked; my ideas for overall appearance. | 3.64 (1.98) | 4.07 (1.68) |
| 2 | How I really feel about people that I work for, or work with | 3.61 (1.74) | 4.59 (1.82) |
| 3 | The thinks I regret as describable for a man to be- what I look for in a man | 3.71 (1.90) | 3.77 (1.73) |
| 4 | Whether or not I owe money, if so how much | 2.35 (1.74) | 3.07 (2.11) |
| 5 | My favorite foods, the ways I like food prepared and my food dislikes | 5.53 (1.37) | 5.00 (1.56) |
| 6 | What it takes to hurt my feelings deeply. | 3.50 (2.20) | 3.41 (2.22) |
| 7 | How I feel about the choice of career that I have made- whether or not I am satisfied with it. | 4.28 (2.29) | 4.48 (1.55) |
| 8 | My personal standards of beauty and attractiveness in women-what I consider to be attractive in a woman | 4.07 (2.19) | 4.41 (1.59) |
| 9 | My likes and dislikes in music. | 5.89(1.06) | 5.70 (1.65) |
| 10 | The kinds of things that make me feel especially proud of myself, elated, full of self esteem or self respect. | 4.5 (2.13) | 5.03 (1.53) |
| 11 | My feelings about different parts of my body- legs, hips, weight, chest or bust etc. | 2.35 (1.76) | 3.11 (2.08) |
| 12 | Whether or not I have savings and the amount of my savings. | 2.25 (1.95) | 2.44 (1.88) |
| 13 | What it takes to get me real worried, anxious and afraid. | 3.32 (1.88) | 3.14 (1.95) |
| 14 | My ambitions and goals In my work. | 4.28 (2.29) | 5.07 (1.66) |
| 15 | My personal views on sexual morality- how I feel that I and others ought to behave in sexual matters. | 3.92 (2.12) | 3.96 (1.99) |
| 16 | Whether or not others owe me money; the amount they owe me and who owes it to me. | 2.61 (2.13) | 2.85 (2.07) |
| 17 | Whether or not I now have any health problems e.g.- trouble with sleep, digestion, heart condition, allergies, headaches, piles etc. | 2.82 (1.63) | 2.96 (1.78) |
| 18 | The kinds of movies that I like to see best; the TV shows that are my favorites. | 5.82 (1.41) | 5.85 (1.41) |
| 19 | My past record of illness and treatment. | 3.07 (2.11) | 2.66 (1.51) |
| 20 | My tastes in clothing. | 4.82 (1.74) | 4.62 (1.66) |
| 21 | My views on the present government- government politics etc. | 4.78 (2.11) | 5.00 (1.94) |
| 22 | How I budget my money- the proposition that goes to necessities, luxuries, etc. | 2.85 (1.75) | 3.03 (1.76) |
| 23 | How I feel that my work is appreciated by others (e.g.- boss, fellow-workers, teacher, husband etc. | 3.96 (1.87) | 3.88 (1.88) |
| 24 | What it takes to get me feeling real depressed and blue. | 2.85 (1.64) | 2.96 (1.65) |
| 25 | My favorite ways of spending more spare time, e.g.- hunting, reading, cards, sports events, parties, dancing etc. | 5.61 (1.59) | 5.62 (1.41) |
| 26 | What I feel are my shortcomings and handicaps that prevent me from working as I would like to. | 3.07 (1.71) | 3.37 (1.80) |
| 27 | My personal opinions and feelings about other religious groups than my own e.g.- Islam, Christianity, Hinduism, etc. | 3.71 (2.40) | 3.41 (2.42) |
| 28 | How I spend my money or would like to spend my money. | 3.11 (2.13) | 3.29 (1.99) |
| 29 | The facts of my present sex life- including knowledge of how I get sexual gratification, any problems that I might have, with whom I have relations, if anybody. | 2.28 (1.90) | 2.25 (1.76) |
| 30 | Whether or not I now make special efforts to keep fit, healthy and attractive, e.g.- diet, exercise, makeup etc. | 3.96 (1.95) | 3.85 (1.83) |

**Path analysis for the effect of Lighting on Willingness to Disclose through the mediation of Self-awareness**

Self-awareness_mean^[[1]](#footnote-1)^ was calculated by averaging the scores on each of the three kinds of self-awareness i.e.- public self awareness, private self awareness and awareness of surroundings. We conducted Path analysis through SPSS AMOS to test if such an indirect mediating effect was indeed present. Figure 1 below shows the result of this analysis.

selfawareness_mean

0.14

0.13

Lighting

Willingness to Disclose

Figure 1 shows the result of Path analysis. It shows the extent of indirect effect of Lighting on Willingness to Disclose through a possible mediating variable i.e.- self-awareness_mean. These three variables are treated as measured variables and are therefore presented in rectangle boxes. Two endogenous variables in this model (i.e.- selfawareness_mean and Willingness to disclose ) have also been provided with error terms (i.e.- E1 and E2). The path coefficient from Lighting to selfawareness_mean is 0.13 and that from selfawareness_mean to Willingness to Disclose is 0.14 as shown in this path diagram.

The model created as shown above was a good fit for our data; χ^2^(1, N=55) = 0.104, p=0.748. Also, the Comparative Fit Index (CFI) and the Tucker-Lewis Index (TLI) were greater than 0.95 and the Root Mean Square Error of Approximation (RMSEA) was smaller than 0.05. The maximum likelihood estimate for the effect of Lighting on self awareness was 0.13 (p=0.151) and that of self awareness on Willingness to disclose was 0.14 (p=0.245) as shown in the figure. Both these estimates were small and insignificant. This indicates that Lighting did not have any indirect effect on Willingness to disclose through its effect on self awareness.

1. Selfawareness_mean was used for Path analysis rather than Public Self awareness as the former satisfies the assumption of normality required for path analysis while the latter does not [↑](#footnote-ref-1)
