# Supplementary material for: Can Lighting Influence Self-Disclosure?
Source: Front Psychol. 2017 Feb 23;8:234. doi: 10.3389/fpsyg.2017.00234 (PMC5322203; doi:10.3389/fpsyg.2017.00234)
Supplement: Supplementary file 2 [file Table_2.DOCX]

**Can lighting influence self-disclosure?**

SUPPLEMENTARY MATERIAL

Table 2. The total and percentage of participants who shared information about themselves on each of the items from both groups in experiment 2 measuring actual disclosure .

| Sr. no. | Questions | Mean (SD) Dim | Mean (SD) Bright |
| --- | --- | --- | --- |
| 1 | How do you wish you looked? | 27 (96%) | 27 (96%) |
| 2 | How do you feel about people you study/work with or work/study for? | 28 (100%) | 28 (100%) |
| 3 | What, according to you, makes a man attractive? | 27 (96%) | 28 (100%) |
| 4 | Do you owe money to anyone? If yet? How much? | 27 (96%) | 27 (96%) |
| 5 | What are your likes and dislikes with respect to food? | 28 (100%) | 28 (100%) |
| 6 | What hurts your feelings deeply? | 28 (100%) | 27 (96%) |
| 7 | What is the career choice that you have made? Are you satisfied with your career choice? | 28 (100%) | 24 (85%) |
| 8 | What according to you, makes a woman attractive? | 28 (100%) | 28 (100%) |
| 9 | What are your likes and dislikes with respect to music? | 27 (96%) | 28 (100%) |
| 10 | What makes you proud of yourself? | 26 (92%) | 27 (96%) |
| 11 | How do you feel about your body in general (e.g.- about your weight, legs, hips, waist, chest or bust etc) | 26 (92%) | 27 (96%) |
| 12 | Do you have savings? If yes, how much? | 24 (85%) | 26 (92%) |
| 13 | What makes you worried, anxious and afraid? | 28 (100%) | 26 (92%) |
| 14 | What are your career ambitions and goals? | 26 (92%) | 25 (895) |
| 15 | What are your personal views on sexual morality (e.g.- how you and others should behave in sexual matters) | 27 (96%) | 26 (92%) |
| 16 | Does anyone owe money to you? If yes, how much? | 25 (89%) | 27 (96%) |
| 17 | Do you have any health problems? If yes, which are they? | 28 (100%) | 26 (92%) |
| 18 | Which are your favorite TV serials and movies? | 28 (100%) | 28 (100%) |
| 19 | Have you suffered from any illnesss or health problems in the past? What did you suffer from? | 27 (96%) | 26 (92%) |
| 20 | What kinds of clothing do you like? | 28 (100%) | 28 (100%) |
| 21 | What are your views on the present government (e.g.- the government policies)? | 25 (89%) | 28 (100%) |
| 22 | How do you spend your money? i.e.- proportion that I spend on necessities, luxuries, etc. | 27 (96%) | 28 (100%) |
| 23 | How is your work appreciated by others (teachers, classmates, colleagues etc)? | 28 (100%) | 28 (100%) |
| 24 | What makes you depressed? | 26 (92%) | 24 (85%) |
| 25 | What are your favorite ways of spending spare time/hobbies? | 28 (100%) | 28 (100%) |
| 26 | What are your shortcomings that prevent you from getting ahead in your studies/work? | 26 (92%) | 26 (92%) |
| 27 | What are your personal opinions and feelings about other religious groups other than your own (e.g.- Hindus, Muslims, Christians, Jains, etc) | 25 (89%) | 27 (96%) |
| 28 | How would you like to spend money? | 25 (89%) | 28 (100%) |
| 29 | Are you sexually active? How many sexual partners have you had? | 26 (92%) | 24 (85%) |
| 30 | What special efforts do you make to keep yourself physically healthy and fit? | 27 (96%) | 28 (100%) |
