# Supplementary material for: Can Lighting Influence Self-Disclosure?
Source: Front Psychol. 2017 Feb 23;8:234. doi: 10.3389/fpsyg.2017.00234 (PMC5322203; doi:10.3389/fpsyg.2017.00234)
Supplement: Supplementary file 3 [file Table_3.docx]

**Can lighting influence self-disclosure?**

SUPPLEMENTARY MATERIAL

List of Items used in the Feedback form to be rated on a 7-point rating scale:

1. I felt anonymous during the task. (Anonymity Item 1)
2. While doing the task, I felt that my identity would not be known. (Anonymity Item 2)
3. While doing the task, I was keenly aware of everything in my environment. (Environmental Self Awareness)
4. While doing the task, I was concerned about what other people might think about me.(Public Self Awareness)
5. While doing the task, I was aware of my innermost thoughts. (Private Self Awareness)
6. I felt at ease inside this room.(Affective experience Item1)
7. I felt comfortable inside this room.(Affective experience Item2)
8. This room gave me a pleasant feeling.(Affective experience Item3)
9. While doing the task, I felt a threat to my privacy and social image. (Perceived Threat)
10. I felt constricted inside this room. (Spaciousness Item1)
11. I felt confined inside this room. (Spaciousness Item2)
