# Supplementary figures and images for: Can Lighting Influence Self-Disclosure?
Source: Front Psychol. 2017 Feb 23;8:234. doi: 10.3389/fpsyg.2017.00234 (PMC5322203; doi:10.3389/fpsyg.2017.00234)

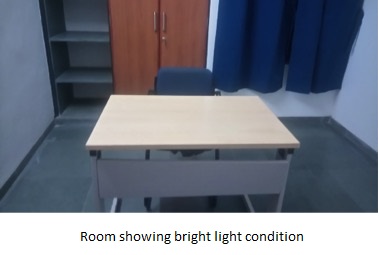

Supplement: Supplementary file 4 [file Image_1.jpeg]

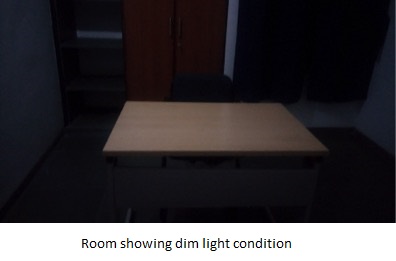

Supplement: Supplementary file 5 [file Image_2.jpeg]
